# RNA-based regulation in type I toxin–antitoxin systems and its implication for bacterial persistence

**DOI:** 10.1007/s00294-017-0710-y

**Published:** 2017-05-30

**Authors:** Bork A. Berghoff, E. Gerhart H. Wagner

**Affiliations:** 1 Institut für Mikrobiologie und Molekularbiologie, Justus-Liebig-Universität, 35392 Giessen, Germany; 2 Department of Cell and Molecular Biology, Uppsala University, 75124 Uppsala, Sweden

**Keywords:** Toxin–antitoxin, Antisense RNA, 5′ UTR structure, Persistence, Depolarization, SOS response

## Abstract

Bacterial dormancy is a valuable survival strategy upon challenging environmental conditions. Dormant cells tolerate the consequences of high stress levels and may re-populate the environment upon return to favorable conditions. Antibiotic-tolerant bacteria—termed persisters—regularly cause relapsing infections, increase the likelihood of antibiotic resistance, and, therefore, earn increasing attention. Their generation often depends on toxins from chromosomal toxin–antitoxin systems. Here, we review recent insights concerning RNA-based control of toxin synthesis, and discuss possible implications for persister generation.

## Introduction

Every organism’s future is unwritten and to a large extent unpredictable. We—as human beings—are aware of this unpleasant fact and try to safeguard ourselves by sanitary and monetary protection. Even though simple organisms like bacteria are not “aware” of the inevitable risks of life, they have inherited genetic programs that have ensured their survival in the past and will do so in the future. Generating phenotypic heterogeneity in a clonal population of bacteria is considered a successful survival strategy, often referred to as bet-hedging (Veening et al. 2008). For example, most bacteria generate subpopulations of non-growing (i.e., dormant) cells that can withstand unfavorable environmental conditions (Lennon and Jones 2011). According to the “microbial scout” hypothesis, cells leave dormancy stochastically to sample the environment (Buerger et al. 2012; Sturm and Dworkin 2015). If conditions are favorable, these pioneering cells can re-populate the environment. Even the smallest subpopulation that rides out a catastrophe can ensure continuity of the bacterial species as such. In line with this concept, pathogenic bacteria generate multidrug-tolerant phenotypic variants that have been denoted persisters due to their ability to survive antibiotic treatment (Bigger 1944). Persisters regularly cause relapsing infections and are considered a major risk to public health (Lewis 2010). In contrast to resistant cells, persisters are unable to multiply in the presence of antibiotics, but rather reside in a dormant state which renders them tolerant towards the action of most antibiotics. Multiple pathways by which persisters arise have been described, most often ultimately resulting in slowed down growth via corruption of essential cellular processes. For example, persistence can be triggered by ATP depletion (Conlon et al. 2016; Shan et al. 2017), nutrient shifts, and metabolic perturbations (Amato et al. 2013; Amato and Brynildsen 2014; Radzikowski et al. 2016), stochastic induction of (p)ppGpp (Maisonneuve et al. 2013; Germain et al. 2015), or indole-activated stress responses (Vega et al. 2012). Distinct pathways might be either essential or rather negligible for persister formation, depending on the experimental/environmental conditions. For example, it was recently challenged whether (p)ppGpp-activated pathways are the dominant source of persister cells (Chowdhury et al. 2016; Shan et al. 2017), and clearly, more experiments are needed to understand the complex nature of persister formation. A recurrent scheme for inducing the persistent state involves toxins from chromosomal toxin–antitoxin (TA) systems (e.g., Dörr et al. 2010; Kim and Wood 2010; Maisonneuve et al. 2011). In unstressed cells, antitoxins normally inhibit either translation or activity of their toxin counterparts. However, when stress occurs, the inhibiting effect is released and cellular processes are impeded by the action of one or several toxins. The different TA systems are classified according to the specific mechanism by which the antitoxin inhibits the toxin directly, or its synthesis. In total, six different TA system types have been described so far (reviewed in Page and Peti 2016). Translational repression of toxin mRNA by an antisense RNA (type I) and inhibition of toxin activity by an antitoxin via protein–protein interaction (type II) are the predominant mechanisms. The first persistence-related toxin gene was *hipA* in *Escherichia coli* (Moyed and Bertrand 1983). HipA belongs to the type II TA system HipAB. Its mode of action was recently deciphered: HipA phosphorylates glutamyl-tRNA synthetase, causing uncharged tRNA accumulation, thereby triggering the synthesis of the alarmone (p)ppGpp (Germain et al. 2013, 2015; Kaspy et al. 2013). Accumulation of (p)ppGpp results in activation of Lon protease, which, in turn, activates several toxins from type II TA systems via degradation of the cognate antitoxins. Most of the Lon-activated toxins are RNA endonucleases that corrupt translation, induce growth arrest, and lead to persister formation (Maisonneuve et al. 2011, 2013). Moreover, some RNA endonucleases were reported to impact stress responses and biofilm formation (Wang and Wood 2011), or to attack phage mRNAs to obstruct phage propagation (Otsuka 2016). Type II TA systems have intensively been studied with regard to the particular mode of toxin action, the regulatory interplay between the toxin–antitoxin partners, and the implications for persister formation (see Gerdes and Maisonneuve 2012; Maisonneuve and Gerdes 2014; Page and Peti 2016; Rocker and Meinhart 2016 for recent reviews). In this review, we will discuss the peculiarities of type I TA systems. Most toxins of type I TA systems are small hydrophobic peptides (<60 amino acids), some of which target the inner membrane to cause depolarization and ATP depletion. This entails inhibition of major cellular processes which is believed to induce persister formation (Unoson and Wagner 2008; Dörr et al. 2010; Verstraeten et al. 2015; Berghoff et al. 2017). Based on current knowledge, we will present new concepts regarding regulation of type I TA systems and bacterial persistence.

## How to keep toxins at bay: the *hok/sok* paradigm

**Fig. 1:**
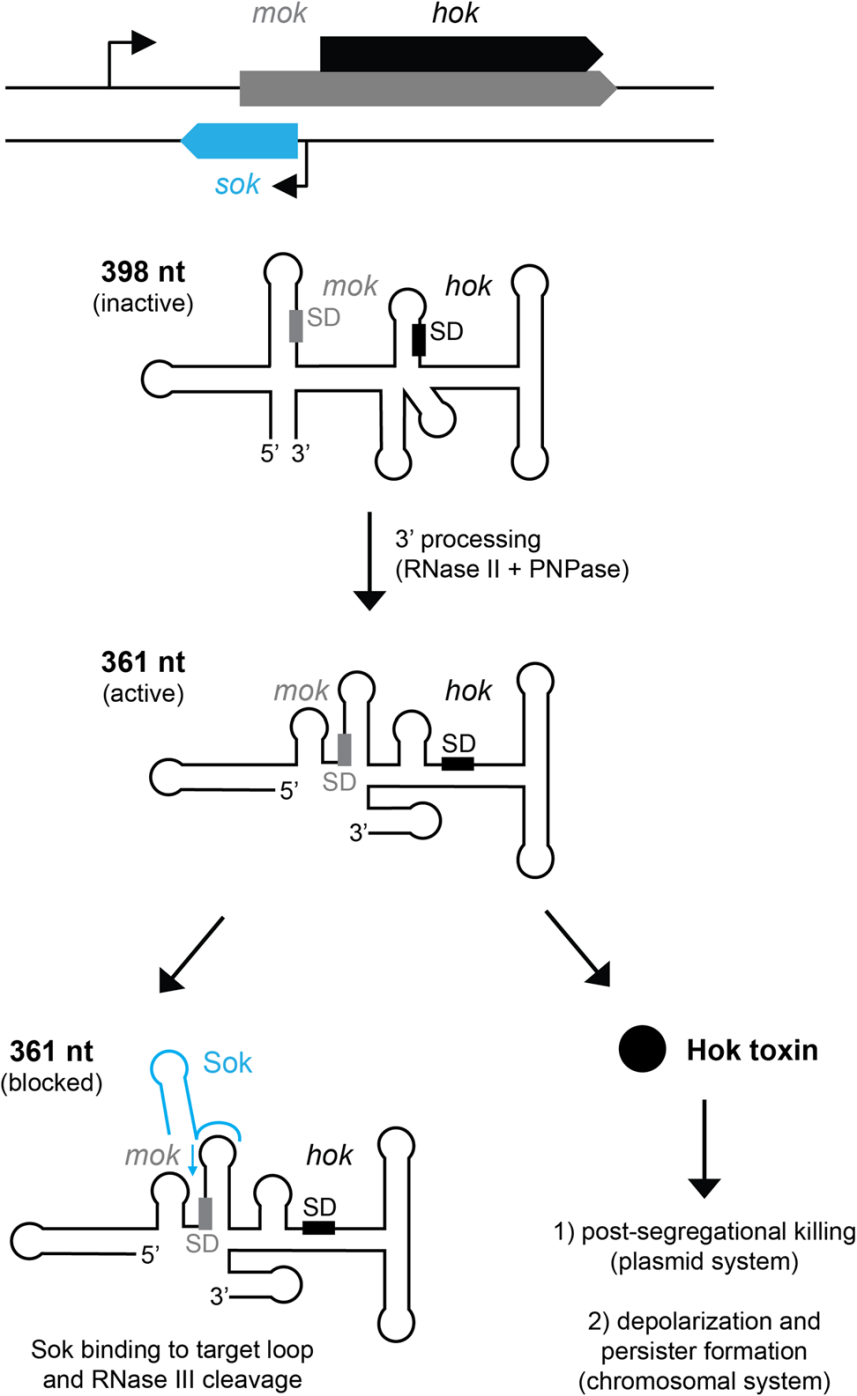
Synthesis of Hok toxins is controlled by two regulatory RNA elements. The arrangement of the *hok/sok* gene locus is depicted in the *upper part of the figure*. *Arrows* indicate promoters. The primary *mok*–*hok* mRNA (398 nt) is translationally inert due to inhibitory secondary structures. 3′ processing generates a translationally active mRNA (361 nt), that is either inhibited by the RNA antitoxin Sok or translated into Hok toxin. Sok binding initiates at the target loop, and the RNA duplex that subsequently forms (indicated by *blue arrow*) is cleaved by RNase III. Plasmid-borne systems are implicated in post-segregational killing, and chromosomal systems contribute to bacterial persistence. *SD* Shine-Dalgarno sequence. Figure based on Gerdes and Wagner (2007). See text for details

Type I toxin–antitoxin systems were initially discovered in bacterial plasmids as post-segregational killing (PSK) systems. Such loci, exemplified by its founding member *hok/sok* on plasmid R1, confer stable plasmid maintenance by killing cells that have lost the plasmid (Gerdes et al. 1986; Weaver and Tritle 1994). The unstable antitoxin RNA is rapidly purged from plasmid-free progeny cells, leading to uninhibited translation of the stable toxin mRNA; the toxin causes cell death through membrane damage. Killing makes sense from the point of view of plasmid maintenance, but for chromosomally encoded TA systems, less is known on whether toxicity plays out as killing or growth arrest/retardation. Clearly, in plasmid-containing cells, toxicity must be tightly controlled. The same holds true for chromosomally encoded type I TA systems which were initially identified as homologues of their plasmid counterparts like *hok/sok* (Gerdes et al. 1986). The *E. coli* K-12 genome encodes five *hok*/*sok* homologues (Pedersen and Gerdes 1999), and a recent study implicates one of these systems (*hokB/sokB*) in membrane depolarization and persister formation (Verstraeten et al. 2015). Regulation of *hok* expression has thoroughly been studied in the plasmid-borne system (reviewed in Gerdes and Wagner 2007), and the same regulatory principles are expected to apply to the chromosomal ones. Translation of *hok* (“host killing”) depends on translation of the overlapping upstream reading frame *mok* (“modulation of killing”). The primary full-length *mok*–*hok* transcript (398 nt) is stable and translationally inert due to structural sequestration. However, slow processing by RNase II and polyribonucleotide nucleotidyltransferase (PNPase) removes 39 nt from the 3′ end and induces structural rearrangements, resulting in a translationally active mRNA (361 nt; Fig. 1). In plasmid-containing cells, the RNA antitoxin Sok (“suppressor of killing”) binds to an accessible U-turn structure in the target loop of the 361-nt-long transcript and represses *mok*, and consequently *hok* translation (Fig. 1). The RNA heteroduplex is cleaved by RNase III and then decays. Thus, inhibition of Hok translation depends on two regulatory RNA elements: an inhibitory secondary structure in the inert *mok*–*hok* transcript and the antitoxin Sok. This is reminiscent of other chromosomal type I TA systems found in *E. coli*, and two-layer control might represent a general regulatory feature of some of these systems.

## SOS induced toxins: TisB and DinQ

**Fig. 2:**
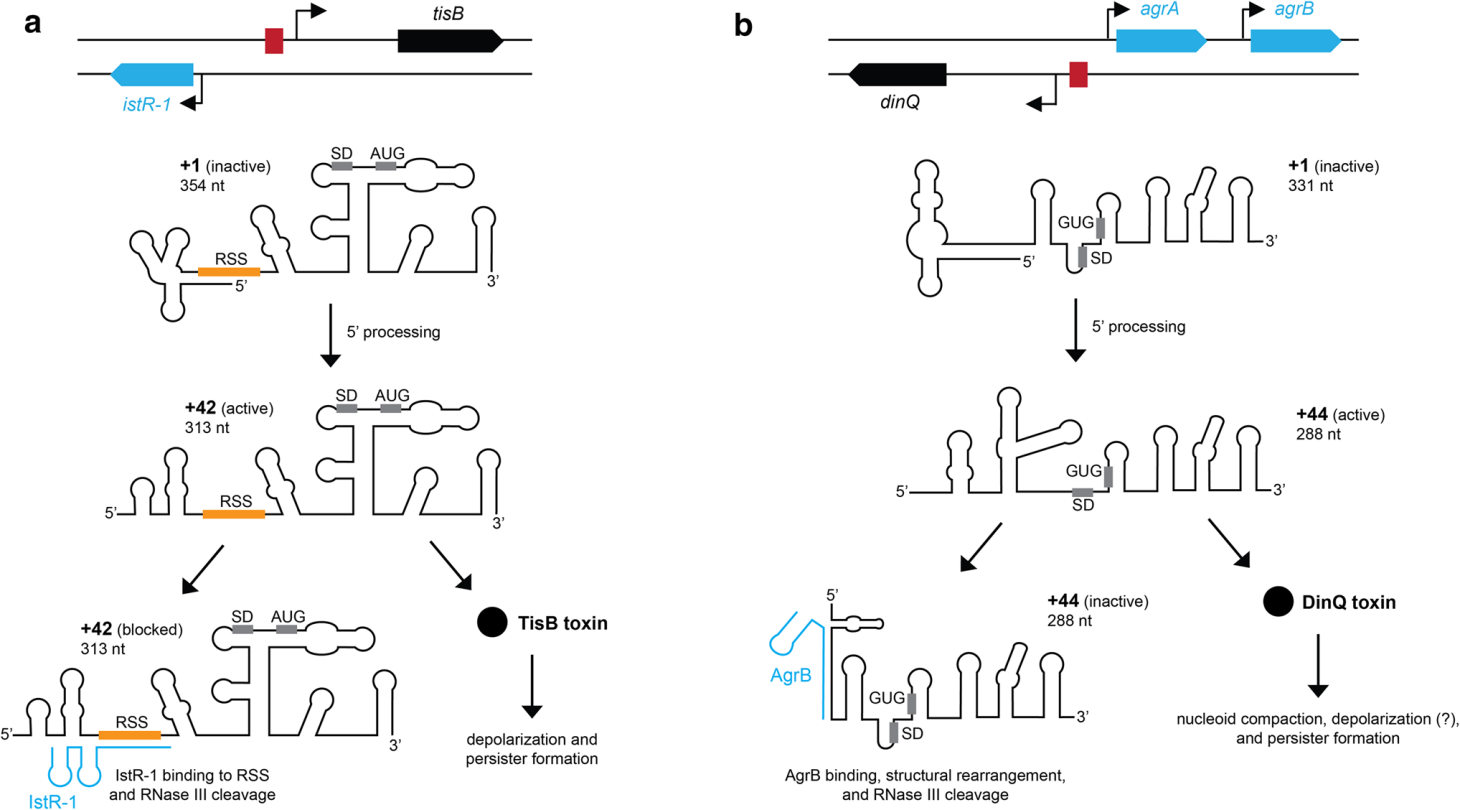
RNA-based regulation of the SOS-responsive toxins TisB and DinQ. The loci for *tisB/istR*-*1* (**a**) and *dinQ/agrAB* (**b**) are depicted in the *upper part* of the figure. *Arrows* indicate promoters and *red boxes* represent binding sites for LexA. In both cases, primary mRNAs (+1) are translationally inert due to secondary structures that prevent ribosome binding. 5′ processing generates translationally active mRNAs (*tisB* +42 or *dinQ* +44) with accessible sites for ribosome loading. The active mRNAs are either inhibited by their cognate RNA antitoxins, or translated into toxin. Both TisB and DinQ are implicated in persister formation under SOS conditions. AUG/GUG: start codon; *SD* Shine-Dalgarno sequence, *RSS* ribosome standby site. See text for details

The chromosomally encoded type I TA systems *tisB/istR*-*1* and *dinQ/agrB* in *E. coli* are implicated in persister formation and survival upon DNA damage, respectively (Dörr et al. 2010; Weel-Sneve et al. 2013; Berghoff et al. 2017). In both systems, the toxin and antitoxin genes are transcribed divergently in a non-overlapping fashion. While the antitoxins are constitutively expressed, transcription of the toxin genes is controlled by LexA, the master regulator of the response to DNA damage (SOS response) (Fig. 2). Under non-stress conditions, LexA binds to the so-called LexA boxes within the promoter region of SOS genes and represses transcription. LexA box sequences are classified by their “heterology index” (HI), indicating how tightly the LexA repressor binds to the particular sequence: a low HI indicates tight repression (Lewis et al. 1994). It makes sense that transcriptional control by LexA repression is complemented by inhibition at the post-transcriptional level, because transcriptional off-states are intrinsically difficult to obtain (Golding et al. 2005; Levine and Hwa 2008). Indeed, leaky transcription is observed for both *tisB* (Berghoff et al. 2017) and *dinQ* (Weel-Sneve et al. 2013), even though both genes have comparably low HI indices of 1.81 and 3.92, respectively (Courcelle et al. 2001). Maintaining a stable off state during normal growth involves two RNA elements. In the case of *tisB*, one is an inhibitory 5′ UTR structure that masks a ribosome standby site (RSS) in the +1 transcript to prevent translation (Darfeuille et al. 2007). A processing event generates the translationally active +42 mRNA, in which the RSS is accessible for ribosome preloading (Fig. 2a). A scarless chromosomal deletion of the first 41 nucleotides of *tisB* mRNA (Δ1-41) shows leaky expression of the translationally active +42 mRNA, and consequently, inappropriate TisB synthesis and depolarization under conditions of low DNA damage (Berghoff et al. 2017). Since binding of the antitoxin IstR-1 to the +42 mRNA triggers RNase III cleavage and thereby removes +42 transcripts, the effect on depolarization is potentiated when IstR-1 is simultaneously deleted. These results demonstrate that an additional regulatory element (the inhibitory 5′ UTR structure) acts to complement the antitoxin RNA for tight control of toxin expression.

The *dinQ/agrB* system shares many similarities with *tisB/istR*-*1* with respect to post-transcriptional regulation: the *dinQ* +1 transcript is translationally inactive and has to be processed at its 5′ end to produce the translationally active +44 transcript. Translation of +44 mRNA is repressed by binding of the antitoxin AgrB (Weel-Sneve et al. 2013; Kristiansen et al. 2016) (Fig. 2b). There are, however, differences between the *dinQ/agrB* and *tisB/istR*-*1* systems: (1) translation of *dinQ* +44 mRNA does not rely on ribosome standby, (2) AgrB does not directly compete with ribosome binding, but rather induces structural rearrangements to sequester the Shine-Dalgarno (SD) sequence, and (3) the primary function of DinQ toxin might not be depolarization of the inner membrane (Fig. 2b). Although plasmid-borne overexpression of DinQ causes depolarization (Weel-Sneve et al. 2013), deletion of *dinQ* does not affect depolarization upon ciprofloxacin treatment, and yet, persistence is impeded (our unpublished data). By contrast, deletion of *tisB* abolishes depolarization, and TisB, therefore, appears to be the main factor for membrane depolarization under SOS conditions (Berghoff et al. 2017). DinQ may primarily regulate nucleoid compaction during repair of DNA lesions, since moderate overexpression of DinQ (by *agrB* deletion) leads to an extended period of nucleoid compaction (Weel-Sneve et al. 2013). The reduced survival of the *agrB* deletion strain upon UV stress further demonstrates that DinQ levels need to be tightly controlled, and that the regulatory RNA elements play important roles in this regard.

## Further toxin mRNAs with regulatory elements in their 5′ UTRs: *zorO* and *shoB*

Besides *tisB/istR*-*1* and *dinQ/agrB*, two additional type I TA systems display a divergent gene orientation and antitoxin RNA-binding sites far upstream of the ribosome-binding site (RBS) of the toxin mRNA. In *E. coli* O157:H7 (EHEC), the *zorO*-*orzO* locus encodes the ZorO toxin and the antisense RNA OrzO. OrzO counteracts ZorO toxicity by base pairing to the mRNA (Wen et al. 2014). The primary *zorO* mRNA supports little translation due to sequestration of the RBS in a stem. As in the case of *tisB*, 5′ processing generates a translationally active transcript: removal of the 5′-most 28 nucleotides causes a structure change, so that ribosome preloading at a single-stranded RSS can occur (Wen et al. 2016). The OrzO RNA competes with ribosome standby and triggers RNase III cleavage. ZorO is a small hydrophobic peptide (29 amino acids) that may contribute to regulating cellular growth in response to nutrient shifts (Wen et al. 2014).

The *shoB*–*ohsC* locus was identified in a screen for small RNAs in *E. coli* (Kawano et al. 2005) and subsequently shown to constitute a bona fide type I TA pair: ShoB toxicity can be abolished by OhsC expression (Fozo et al. 2008). Full-length (~320 nt) and 5′ processed (~280 nt) *shoB* transcripts were identified, but again, only the processed mRNA was translationally active (Fozo et al. 2008). The regulatory elements within *shoB* mRNA have not yet been studied in detail, but likely involves secondary structures that inhibit translation and need to be resolved by 5′ processing. The small hydrophobic ShoB toxin (26 amino acids) causes depolarization upon overexpression (Fozo et al. 2008). It will be exciting to see whether and—if so—under which conditions ShoB and ZorO contribute to bacterial persistence, and whether depolarization is involved.

## Two-layer control of toxin translation blurs the definition of antitoxins

Why are many type I toxin mRNAs endowed with inhibitory RNA elements in their 5′ UTRs? One shared property is that, as for *hok* and *tisB* mRNAs, translation is not coupled to transcription. That is, mRNAs first accumulate in a translationally inactive form, and toxin synthesis only occurs after processing. Here, the RNA antitoxin alone is not sufficient to tightly block toxin expression, as supported by recent results with the *tisB/istR*-*1* system. Deletion of the *tisB* 5′ UTR structure triggers depolarization even without strong SOS induction and in the presence of the antitoxin IstR-1 (Berghoff et al. 2017). Most intriguingly, persister levels of Δ*istR* and Δ1-41 single deletion strains were increased by 4- and 11-fold, respectively, in comparison to the wild type upon ciprofloxacin treatment (Berghoff et al. 2017). The 5′ UTR structure seems, in our experimental setting, to be more important for controlling TisB-dependent persister formation than the antitoxin RNA. This can be interpreted as control by two antitoxin elements: the intrinsic antitoxin (toxin mRNA 5′ structure) and the extrinsic antitoxin (antisense RNA). Importantly, the “two antitoxins” act sequentially, which causes a delay in toxin synthesis. As long as the intrinsic 5′ UTR structure is present in the toxin mRNA, no translation occurs, and binding of the antisense RNA only plays a minor role. If the 5′ UTR structure is resolved by processing, the antisense RNA takes over, and mRNA decay irreversibly removes translationally active transcripts from the cell. The frequency of processing and the abundance of extrinsic RNA antitoxin, therefore, determine whether and when toxin is produced. Consequently, using “two antitoxins” for regulation generates a continuum of toxin-producing cells in terms of time and toxin abundance. This phenotypic heterogeneity likely provides an adequate rate of persister cell formation when stress occurs. Some cells may quickly produce high amounts of toxins and immediately enter the persistent state, aiding the survival of the population in case of fatal stress. Other cells may instead continue to grow first and turn into persisters later, which might be beneficial in case of mild but enduring stress. Hypothetically, upon return to non-stress conditions, the slow responder cells might not enter the persistent state at all and instead proliferate.

When wild-type *E. coli* cells were pre-treated with low ciprofloxacin concentrations (1× MIC, minimal inhibitory concentration) and subsequently challenged with a high dose of ciprofloxacin (100× MIC), persister levels were enhanced by ~tenfold compared to samples without pre-treatment (Berghoff et al. 2017). Importantly, deleting “both antitoxins” in the *tisB/istR*-*1* system (Δ1-41 Δ*istR*) causes a strong increase in persister levels upon ciprofloxacin treatment (>100-fold compared to the wild type), irrespective of pre-treatment. The TA system behaves as if it changed from an inducible (wild-type situation) to a stochastic mode (by deletion of both regulatory RNA elements). Assuming that the wild-type situation confers maximal fitness to *E. coli* in its natural habitats, our observations suggest two main conclusions: (1) type I TA systems, e.g., *tisB/istR*-*1*, are “built” to be silent under non-stress conditions, and (2) tight regulation by two antitoxin elements provides an adequate fraction of persisters upon environmental stress. A similar rationale would likely also apply to *hokB/sokB*, *dinQ/agrB*, *zorO/orzO*, and *shoB/ohsC*. Competition experiments between wild-type and antitoxin-deletion strains with regard to persistence might answer these questions in the future.
